# Sleeping Sites and Latrines of Spider Monkeys in Continuous and Fragmented Rainforests: Implications for Seed Dispersal and Forest Regeneration

**DOI:** 10.1371/journal.pone.0046852

**Published:** 2012-10-08

**Authors:** Arturo González-Zamora, Víctor Arroyo-Rodríguez, Ken Oyama, Victoria Sork, Colin A. Chapman, Kathryn E. Stoner

**Affiliations:** 1 División de Posgrado, Instituto de Ecología A.C., Xalapa, Veracruz, México; 2 Centro de Investigaciones en Ecosistemas, Universidad Nacional Autónoma de México (UNAM), Morelia, Michoacán, México; 3 Escuela Nacional de Estudios Superiores Unidad Morelia, Universidad Nacional Autónoma de México (UNAM), Morelia, Michoacán, México; 4 Department of Ecology and Evolutionary Biology, University of California Los Angeles, Los Angeles, California, United States of America; 5 McGill School of Environment and Department of Anthropology, McGill University, Montreal, Quebec, Canada; 6 Wildlife Conservation Society, Bronx, New York, United States of America; 7 Department of Biological and Health Sciences, Texas A & M University-Kingsville, Kingsville, Texas, United States of America; Institut Pluridisciplinaire Hubert Curien, France

## Abstract

Spider monkeys (*Ateles geoffroyi*) use sites composed of one or more trees for sleeping (sleeping sites and sleeping trees, respectively). Beneath these sites/trees they deposit copious amounts of dung in latrines. This behavior results in a clumped deposition pattern of seeds and nutrients that directly impacts the regeneration of tropical forests. Therefore, information on the density and spatial distribution of sleeping sites and latrines, and the characteristics (i.e., composition and structure) of sleeping trees are needed to improve our understanding of the ecological significance of spider monkeys in influencing forest composition. Moreover, since primate populations are increasingly forced to inhabit fragmented landscapes, it is important to assess if these characteristics differ between continuous and fragmented forests. We assessed this novel information from eight independent spider monkey communities in the Lacandona rainforest, Mexico: four continuous forest sites and four forest fragments. Both the density of sleeping sites and latrines did not differ between forest conditions. Latrines were uniformly distributed across sleeping sites, but the spatial distribution of sleeping sites within the areas was highly variable, being particularly clumped in forest fragments. In fact, the average inter-latrine distances were almost double in continuous forest than in fragments. Latrines were located beneath only a few tree species, and these trees were larger in diameter in continuous than fragmented forests. Because latrines may represent hotspots of seedling recruitment, our results have important ecological and conservation implications. The variation in the spatial distribution of sleeping sites across the forest indicates that spider monkeys likely create a complex seed deposition pattern in space and time. However, the use of a very few tree species for sleeping could contribute to the establishment of specific vegetation associations typical of the southeastern Mexican rainforest, such as *Terminalia-Dialium*, and *Brosimum-Dialium*.

## Introduction

There is ample evidence that several mammal species defecate in latrines [1]. This behavior is often related to olfactory communication among individuals or groups as part of their reproduction, territory marking, and resource defense [2], [3]. This is evident in several carnivore [4], [5] and primate species [2], [6], [7]. Nevertheless, in some cases latrines are simply the result of animal social behaviors leading to aggregation (e.g., lek formation, repeated perch use, and sleeping sites [8], [9], [10]). Regardless of the function that latrines serve for animals, it is increasingly recognized that latrines may have critical ecological consequences, especially those of frugivorous species, as latrines result in a clumped deposition pattern of seeds for many species (e.g., primates, *Alouatta seniculus:* [6]; *Lagothrix lagothricha and A. seniculus:* [11]; *Alouatta caraya:* [12]; rhinoceros, *Rhinocerus unicornis:* [13] *;* badgers, *Meles meles:* [14] *;* tapirs, *Tapirus terrestris:* [15] *;* elephants, *Loxodonta africana and Elephas maximus*: [16]) and are accompanied by a large amount of dung and nutrients [17], [18], [19]. Therefore, as a form of spatially contagious seed dispersal (*sensu* [20]), latrines may affect the recruitment, spatial distribution, abundance, and regenerative potential of plant populations, directly impacting vegetation dynamics [7], [20], [21], .

The impact of latrines on plant assemblages may be particularly relevant in highly frugivorous mammals, such as spider monkeys (*Ateles* spp.) [23], [24], [25], [26]. These Neotropical primates live in social systems with a high degree of fission–fusion dynamics, adjusting their subgroup size to local food availability [27]. Individuals forage in different subgroups during the day, whereas they regularly form larger subgroups in the evening congregating in sites (sleeping sites, hereafter) composed of one or several closely spaced large trees to sleep (sleeping trees, hereafter) located near available food resources [7], [28], [29]. These sleeping sites can vary in size depending on the number of monkeys using them on a particular day [7], [28]. Thus spider monkey ranging behavior results in a mixed seed deposition pattern, with a fraction of seeds deposited during the day in individual scats distributed across the forest and the remaining seeds deposited at night or early morning in one or more latrines beneath sleeping sites [7], [28]. This mixed seed deposition pattern has important implications for seed dispersal and seedling recruitment, as both deposition patterns may result in different areas of seedling recruitment [7], [22].

Despite the potential importance of latrines of spider monkeys for forest regeneration, to our knowledge no study has assessed the density and spatial distribution of latrines or sleeping sites, nor the composition, preferences, or the characteristics of sleeping trees. Furthermore, because of the serious conservation threat to spider monkeys, they are increasingly forced to inhabit fragmented forests [25], [30], where both the availability of food resources and large trees are scarce [31], [32]. Thus, it is necessary to evaluate if the characteristics of sleeping trees and the density and spatial distribution of both sleeping sites and latrines differ between continuous and fragmented forests as this may contribute to the altered tree community dynamics of forest fragments. Overall, this information may have critical ecological and conservation implications for understanding the dynamics of tropical forests [22].

In this paper, we present novel information on the density and spatial distribution of sleeping sites and latrines of spider monkey in continuous and fragmented tropical rainforest in Lacandona, Mexico. We describe the arboreal composition, preferences, and structure of principal sleeping trees used by this species, and assess if these characteristics differ between forest conditions. Because of a lower availability of large trees and a limited home range size in forest fragments [31], [32], [33], we predicted a lower density of sleeping sites and latrines in forest fragments than in continuous forests. Additionally, the spatial distribution of sleeping sites and latrines will be highly variable, depending on the distribution of fruits in space and time [8]. Finally, since the lower availability of fruits in fragments can ‘force’ spider monkeys to spend more time consuming leaves [25], [34], [35] that are more widely available throughout the forest than fruit, we predicted that the inter-latrine distances will be lower in fragments than in continuous forest. Moreover, in forest fragments spider monkeys will use smaller sleeping trees (i.e., with lower diameter at breast height, DBH) from fewer tree species than in continuous forest.

## Materials and Methods

### Ethics Statement

All necessary permits were obtained for the described field studies. This study was also approved by the Mexican Office for the Environment and Natural Resources (SEMARNAT), the Office for the Biological Reserve of Montes Azules (BRMA), and the Consejo Nacional de Ciencia y Tecnología (CONACYT) from Mexico (Projects CB-2005-51043 and CB-2006-56799). Moreover, we conducted this study with the authorization of the owner of forest fragments of the Reforma and Zamora Pico de Oro communities. Since our research involved an observational field study and did not involve any contact with the animals, we met all ethical and legal requirements established by the American Society of Primatologists (ASP), Animal Care and Use Committee, and Ethical Committee of the Zoological Society of London for work on primates.

### Study Area

The Mexican Lacandona rainforest constitutes the southwestern sector of the Maya forest in Mexico, and it is one of the most important rainforest remnants in Mesoamerica [36]. The area is located in the northeastern portion of the state of Chiapas, and is delimited by the Guatemalan border on the south and east, and by the Chiapas Highlands on the north and west. The predominant climate in the region is warm and humid with abundant summer rainfall [37]. Average monthly temperatures range from 24°C to 26°C, and mean annual rainfall is 2500–3500 mm, with roughly 80% of the rains falling between June and November. The area was originally covered by over one million ha of rainforest, of which about half remain today [38], [39].

We worked in two adjacent areas separated by the Lacantún River (>150 m wide): the Marqués de Comillas region (MCR, eastern side of the river) encompassing ca. 176,200 ha of fragmented forest, human settlements, and agricultural lands [40], and the Montes Azules Biosphere Reserve (MABR, western side) comprising ca. 331,000 ha of undisturbed old-growth forest [41]. The original predominant vegetation type is tropical rainforest [42], but human colonization and deforestation of MCR since the 1960s resulted in the rapid disappearance and fragmentation of the forest [40]. Approximately 50% of the land surface of MCR is now used for cattle ranching and agriculture, but several forest fragments (0.5–1500 ha) remain.

We assessed latrines and sleeping sites used by eight independent spider monkey communities: four sites in continuous forest of the MABR separated by at least 5 km, and four sites in different forest fragments (ranging from 17 to 1125 ha) within the MCR (Table 1). We chose these sites because previous studies had been conducted here and we had information on the home ranges of each community (previously identified in a 16-month study [32], [34], [35]). All fragments in MCR were isolated ≥24 yrs ago, are immersed in an anthropogenic matrices (pastures, cocoa plantations, agricultural lands, and rural settlements), and their distances to continuous forest ranged from 200 to 1200 m. The isolation distance among fragments ranged from 50 m to 450 m. Spider monkey communities ranged from 25 to 44 individuals, and their home ranges varied from 32 to 90 ha [32], [34], [35].

**Table 1:** Sites studied in the Lacandona rainforest, Mexico.

Sites	Area (ha)	Coordinates	DNF	DCF	YSF	CS
*Continuous forest*						
CF1	331,000	16°06′25.01″N 90°59′16.61″O	n/a	n/a	n/a	44
CF2	331,000	16°06′08.62′′N 90°58′05.29′′O	n/a	n/a	n/a	–
CF3	331,000	16°06′50.25′′N 90°56′24.46′′O	n/a	n/a	n/a	36
CF4	331,000	16°09′31.84′′N 90°54′17.56′′O	n/a	n/a	n/a	44
*Forest fragments*						
FF1	1,125	16°15′10.83′′N 90°49′53.82′′O	100	1100	27	41
FF2	33	16°16′54.15′′N 90°50′19.91′′O	100	3150	25	25
FF3	30	16°19′54.85′′N 90°51′10.71′′O	450	200	29	35
FF4	35	16°10′51.61′′N 90°52′26.50′′O	50	470	25	20

a DNF = distance to nearest forest fragments; DCF = distance to continuous forest; YSF = years since fragmentation; CS = community size of spider monkeys.

(n/a) not applicable; (–) unavailable data.

### Data Collection

We recorded all sleeping sites, the principal sleeping trees, and latrines located in a continuous 30-ha area of each community’s home range, which totaled 240 ha of sampling area across the eight communities. We performed two surveys in all sites, one during March 2010 (i.e., dry season, with lower availability of fruit sources), and another during August and September 2010 (i.e., rainy season, with higher availability of fruit sources). In each survey, two people (i.e., the first author and an experienced local field assistant) walked slowly and in parallel (separated approximately 5 m) through the entire area looking for latrines. When a tree with a DBH ≥30 cm was located in the trek, we made a careful search in the ground taking into account the surface of tree crown. Depending on weather and terrain conditions, we spent between 2 and 4 days per site.

**Figure 1:**
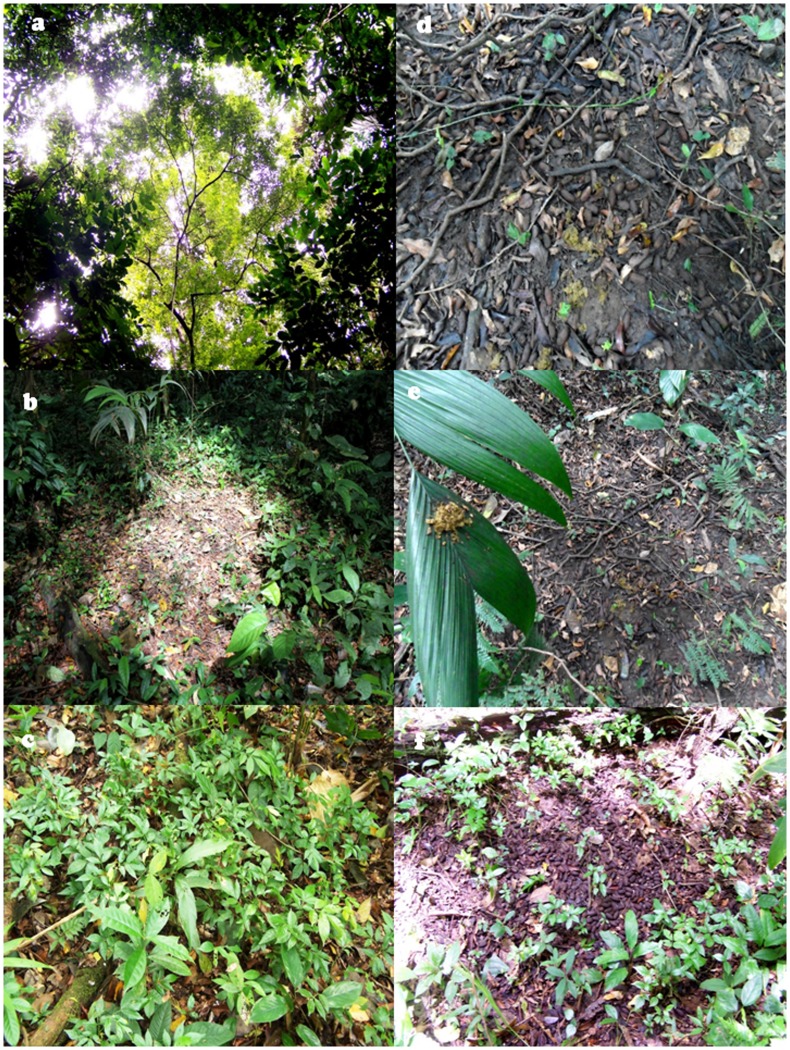
Lateral branches of a *Dialium guianense* sleeping tree (a), and different characteristics of latrines of spider monkeys (*Ateles geoffroyi*) in the Lacandona rainforest, Mexico: form of latrine (b), carpet of seedlings (c), seeds and fresh dung (d), spots of feces on the leaves of understory palms (e), and seeds and seedlings (f).

In general, latrines were easily identified in the field due to their characteristic odor and appearance, which are notably different from those of the feces of the black howler monkeys (*Alouatta pigra*) the other primate species present in the region. In the study area, the latrines are usually located below one of the main lateral branches of a sleeping tree near the crown edge (Figure 1a). They are on the ground and have a semicircular shape, ranging between 1 and 3 m of diameter (Figure 1b). They can be covered by a carpet of new and old multispecies seeds, seedlings, litter, and fresh and old feces (Figures 1c-f). Latrines are also easily recognized by the presence of spots of feces on the leaves of vegetation that are surrounded and superimposed over the latrine (Figure 1e).

After locating a latrine, we positioned in its center (i.e., with higher amount of feces and seeds) and looked perpendicularly for the lateral branch in the canopy that could be used by spider monkeys to sleep and defecate. We recorded the tree species used (sleeping tree), its DBH, and location (with GPS). In the few cases that more than one branch was above the latrine’s location, we assumed that the principal sleeping tree was the one with a branch above the latrine that, through their particular structure (e.g., large and horizontal tree branch bifurcations), could accommodate the individuals to defecate. This assumption was based on qualitative comparisons with the cases in which we only observed one large lateral branch above the latrines (Figure 1a). Although, it is possible that a few individuals slept in other neighboring sleeping trees, they did not form distinct latrines detectable during the field search. This can be possible if the number of individuals was very low and/or if the neighboring sleeping trees were smaller, and hence, the subgroups sleeping in them were smaller than the subgroups sleeping in the tree we recorded.

We also determined the inter-latrine distances with ArcGIS 9.0. This measure was used to identify different sleeping sites. Based on Russo & Augspurger [29], the average (± SD) size of a sleeping site is 89.3±37.4 m^2^. Therefore, we considered all latrines located at ≤10 m among each other as belonging to the same sleeping site. We are confident that this method was accurate in identifying different sleeping sites, as excluding these latrines, the average (± SD) inter-latrine distance was 425±370 m, indicating that they most probably belonged to different sleeping sites [29]. After identifying each sleeping site, we calculated the number of sleeping trees and latrines within each sleeping site.

**Figure 2:**
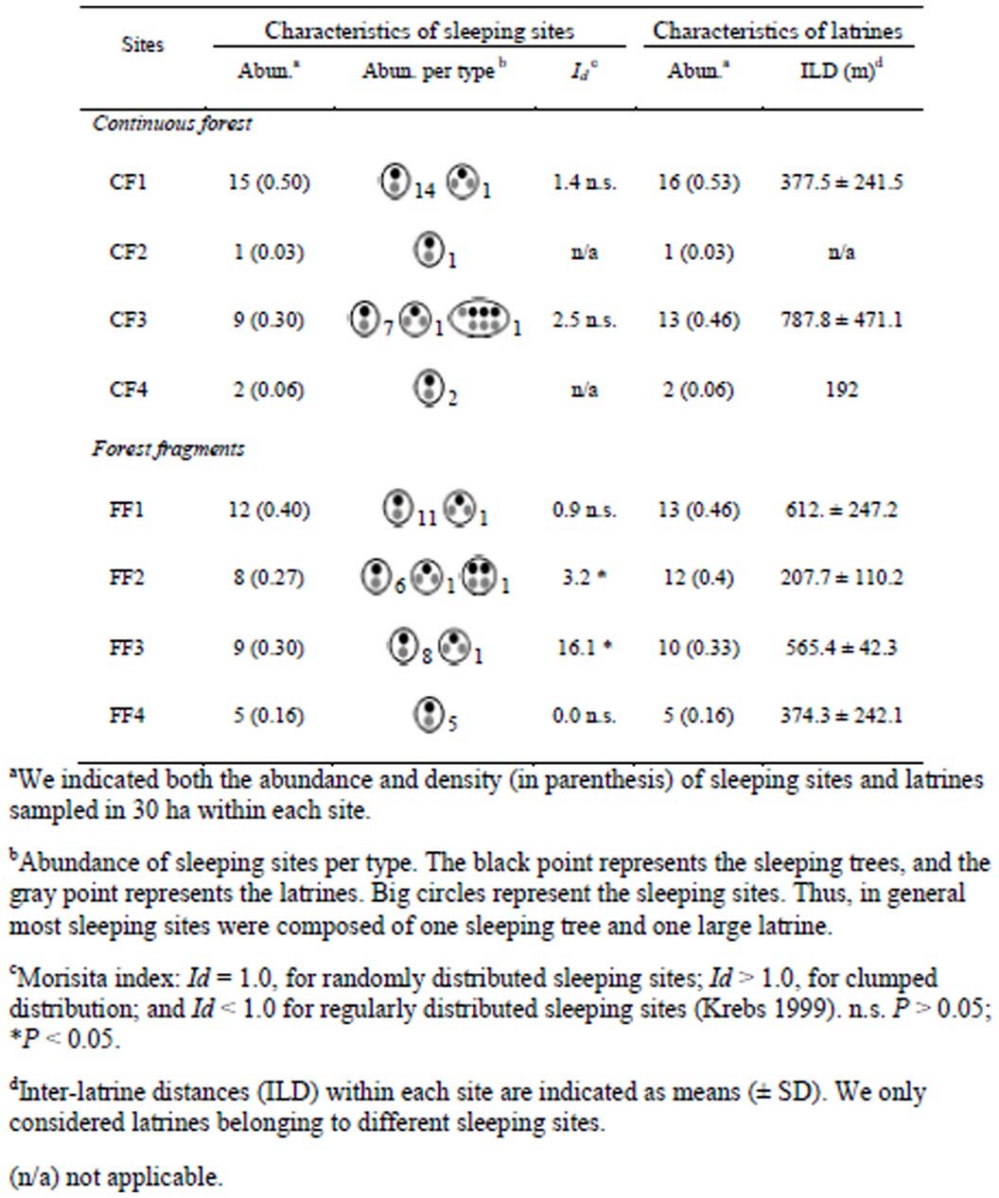
Sleeping sites and latrines of spider monkeys (*Ateles geoffroyi*) in four continuous forest sites and four forest fragments in the Lacandona rainforest, Mexico.

To estimate preferences of monkeys for certain sleeping trees, we also evaluated the density of trees with DBH ≥30 cm in ten 100×2-m plots (0.2 ha) randomly located within each 30-ha sampled area. This vegetation sampling was only performed in the sites with a higher density of sleeping sites and latrines (Figure 2).

### Data Analysis

To test for differences in the density of latrines, species richness, and DBH of sleeping trees, and inter-latrine distances between continuous forest and forest fragments we used analyses of deviance (ANODE) with generalized linear models (GLM). As suggested for count dependent variables (i.e., richness of sleeping tree species), we used a Poisson error and a log link function [43]. However, the differences in density of latrines, DBH of sleeping trees, and inter-latrine distances between both forests conditions were analyzed by used a Normal error and an identity link function, after verifying that the errors of these dependent variables fit normal distributions (Shapiro-Wilk test).

To evaluate the spatial distribution of sleeping sites (i.e., uniform, clumped, or random) within the sampling areas, we plotted in *x* and *y* axes the UTM coordinates of each sleeping site. We divided the 30 ha of sampling area in 1-ha plots and counted the number of sleeping sites that fell within each 1-ha plot. Then, we assessed the distribution pattern of sleeping sites with the Morisita index of dispersion (*I_d_*) [44] using the following formula:



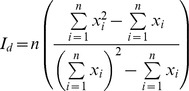
where *n* is the total number of plots in the sample, and *x_i_* is the number of sleeping sites in the *i*-th plot. The value of the index *I_d_* = 1.0 for randomly distributed sleeping sites, >1.0 for clumped sleeping sites and <1.0 for uniformly distributed sleeping sites, ranging from zero to the total number of plots. This index has the advantages of being relatively independent of plot size, density, and sample size [45]. The statistical significance of the departure of each *I_d_* from 1.0 was tested with the statistic χ^2^ (df = *Q* - 1) = 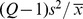 [46], where *Q* in the number of plots in the sample and *s^2^* and 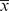 are the variance and mean of the number of latrines per plot in the sample, respectively. As the degree of clumping in nature is frequently strongly influenced by the spatial scale considered [47], we also calculated *I_d_* and the significance of its departure from 1.0 for the whole region, i.e., considering each sampling area as a large plot (n = 8 plots). Thus, we evaluated the spatial distribution of the abundance of sleeping sites across sampling areas.

To analyze the degree to which spider monkeys are selective in their choice of sleeping trees, we used the Manly’s standardized index. This index is based on the selection ratio *w_i_*, which is the proportional use divided by the proportional availability of each resource: *w_i_* = *o_i_*/*π_i_*; where: *o_i_* is the proportion of the sample of used resource units in category *i*, and *π_i_* is the proportion of available resource units in category *i*. Because sampling efforts for “use" versus “availability" were different (30 ha and 0.2 ha, respectively), we calculated the proportional use of each sleeping tree and its proportional availability considering the density of trees per hectare; i.e., number of trees used/30 ha, and number of trees available/0.2 ha. A *w_i_* value larger than 1 indicates a positive selection for the resource (i.e., sleeping trees in our case), and a value less than 1 indicates avoidance of the resource. A value around 1 indicates that the resource was used proportionally to its availability and no selection was noted. The preference/avoidance of each tree species was calculated from the selection ratio *w_i_*, and the statistical significance was assessed with a chi-square test [48]. With this test we compared the observed number of sleeping trees used per species with the expected number under the hypothesis of no selection (i.e., considering that the tree species *i* was used proportionally to its availability) [48]. Additionally, to evaluate if spider monkeys selected larger trees to sleep, for each tree species, we tested for differences in the average DBH of sleeping trees versus the average DBH of the trees available within the home range using Student’s t-tests. In those cases in which we compared a single observation with the mean of a sample, we used the Student’s t-test [49]: t (df = n − 1) = 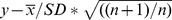; where *y* is the single tree DBH, 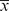 and *SD* are the mean and standard deviation of the trees’ DBH in the sample, respectively and *n* is the number of trees in the sample.

## Results

### Density and Spatial Distribution of Latrines and Sleeping Sites

Overall we found 72 latrines in 61 sleeping sites (Figure 2). Considering the total sampled area (240 ha), the density of latrines and sleeping sites were 0.3 latrines/ha and 0.25 sleeping sites/ha, respectively. The density of latrines did not differ between the continuous forest (0.27 latrines/ha, n = 32 latrines) and forest fragments (0.33 latrines/ha, n = 40 latrines) (GLM, χ^2^ = 0.28, df = 1, *P* = 0.59), nor did the density of sleeping sites differ between the continuous forest (0.23 sleeping trees/ha) and forest fragments (0.28 sleeping trees/ha) (χ^2^ = 0.32, df = 1, *P* = 0.57) (Figure 2).

In general, latrines were uniformly distributed across sleeping sites, as most sleeping sites (89%) had only one latrine beneath one single sleeping tree (Figure 2). Five sleeping sites (8%) had two latrines beneath one single sleeping tree, and these sleeping sites were located in both continuous and fragmented sites. We only found one large sleeping site within the continuous forest with three different sleeping trees and four latrines, and one sleeping site within a forest fragment composed of two different sleeping trees and two latrines (one latrine per sleeping tree) (Figure 2).

**Figure 3:**
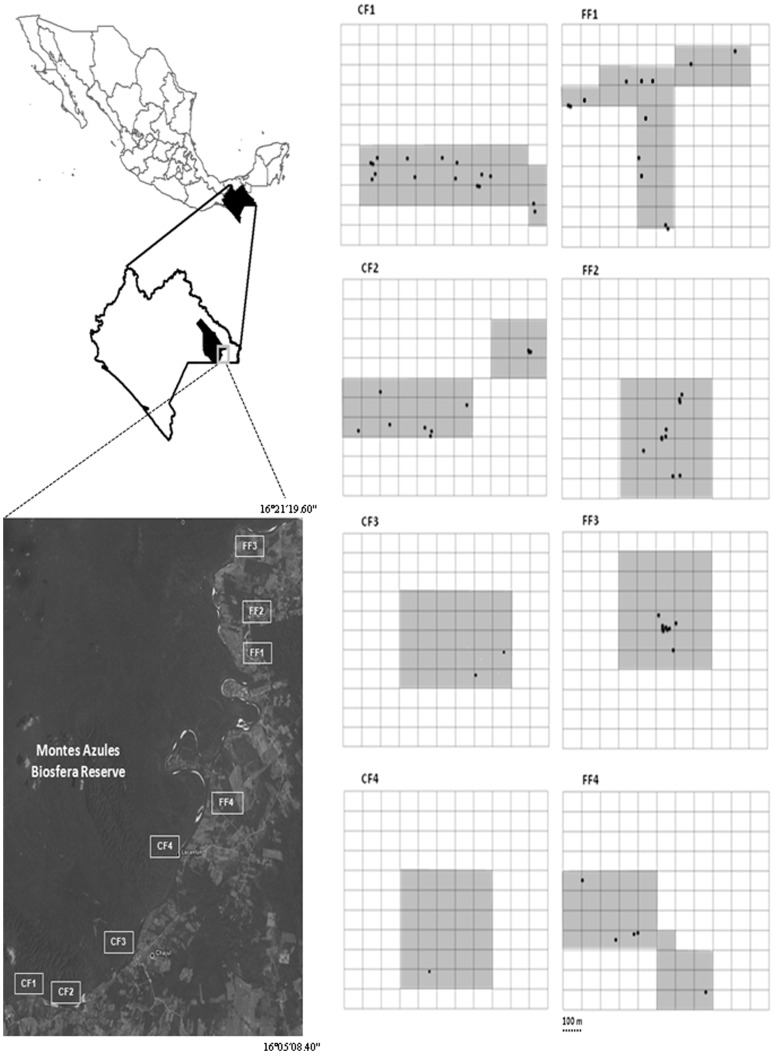
Continuous forest sites and forest fragments studied in the Lacandona rainforest, Mexico. The location of each sleeping site of spider monkeys (dots) within the 30 ha of sampling area (gray shaded areas) area indicated. These areas were divided in 1-ha plots to estimate the spatial distribution of sleeping sites within each study area.

The spatial distribution of sleeping sites within the 30-ha sampled areas was highly variable, being significantly clumped only in two forest fragments, particularly in the smallest one (*Id* = 16.1; χ^2^ = 134.5, df = 29, *P*<0.0001; Figure 2). At a regional scale, sleeping sites showed a clumped distribution (*Id* = 1.23; χ^2^ = 20.97, df = 7, *P* = 0.004), indicating that sleeping sites are particularly abundant in some fragments and areas within the continuous forest, but very scarce in others (Figures 2, 3). Interestingly, the average distance among latrines was almost double in continuous forest (mean ± SD, 585.0±286.7 m) compared to the fragments (296.7±283.6 m), but the difference was not significant (χ^2^ = 1.86, df = 1, *P* = 0.17; Figure 2).

### Tree Species used as Sleeping Sites

The 64 sleeping trees belonged to 9 species, 9 genera, and 9 families (Table 2). There were no significant differences in species richness of sleeping trees between continuous (5 species from 5 families) and fragmented (8 species from 8 families) forests (χ^2^ = 0.04, df = 1, *P* = 0.85). However, the DBH of sleeping trees was two times greater in continuous forest (62.39±36.70 cm) as compared to the forest fragments (35.17±20.75 cm) (χ^2^ = 14.35, df = 1, *P*<0.0001).

**Table 2:** Use and availability of sleeping trees for spider monkeys (*Ateles geoffroyi*) in continuous and fragmented forests in Lacandona, Mexico.^a^

Sites/Families	Species	Treesused	Treesavailable^b^	*w_i_*	DBH used(range, cm)	DBH available^b^(range, cm)	*t*
*Continuous forest*							
CF1							
Meliaceae	*Guarea glabra*	2 (0.07)	4 (20)	0.4	23.3 (17.5–29)	36.2 (30.9–38.8)	−2.91*
Ulmaceae	*Ampelocera hottlei*	4 (0.13)	3 (15)	1.06	41.8 (16.2–55)	63.0 (54.8–76.4)	−1.65n.s
Caesalpiniaceae	*Dialium guianense*	3 (0.1)	4 (20)	0.6	51.3 (27–77)	53.2 (33.7–80.2)	−0.11n.s
Moraceae	*Brosimum alicastrum*	6 (0.2)	1 (5)	4.8	106.3 (52–138)	79.6	−0.96n.s
CF2							
Caesalpiniaceae	*Dialium guianense*	1 (0.03)	–	–	46	–	–
CF3							
Chrysobalanaceae	*Licania platypus*	2 (0.07)	4 (20)	0.67	115.3 (107.6–123)	35.8 (35.5–40.1)	2.02n.s
Caesalpiniaceae	*Dialium guianense*	4 (0.13)	4 (20)	1.35	42.8 (28–51)	61.4 (37.6–95.5)	−1.29n.s
Moraceae	*Brosimum alicastrum*	2 (0.07)	3 (15)	0.90	84.5 (70.3–98.7)	61.0 (54.7–69.7)	1.95n.s
Ulmaceae	*Ampelocera hottlei*	2 (0.07)	2 (10)	1.35	27 (25–29)	33.9 (32.1–35.7)	−2.59n.s
Meliaceae	*Guarea glabra*	1 (0.03)	2 (10)	0.67	24	35.8 (31.5–40.1)	−2.38n.s
CF4							
Caesalpiniaceae	*Dialium guianense*	2	–	–	54.27 (43.3–65.3)	–	–
*Forest fragments*							
FF1							
Caesalpiniaceae	*Dialium guianense*	7 (0.23)	7 (35)	0.72	32.28 (15.0–56.6)	38.6 (30.6–44.2)	−0.91n.s
Moraceae	*Brosimum alicastrum*	3 (0.1)	1 (5)	2.18	31.9 (23.3–38)	70.3	5.77*
Sapotaceae	*Pouteria sp.*	1 (0.03)	–	–	27.7	–	–
Clusiaceae	*Calophyllum brasiliense*	1 (0.03)	–	–	34.8	–	–
FF2							
Moraceae	*Brosimum alicastrum*	1 (0.03)	4 (20)	0.16	42.6	68.5 (54.1–86.6)	1.83n.s
Caesalpiniaceae	*Dialium guianense*	6 (0.3)	1 (5)	4	27.70 (19.4–39)	45.8	2.98*
Meliaceae	*Guarea glabra*	1 (0.03)	1 (5)	0.66	23.5	36	–
Combretaceae	*Terminalia amazonica*	1 (0.03)	–	–	39.5	–	–
FF3							
Meliaceae	*Guarea glabra*	4 (0.13)	1 (5)	2.22	17.6 (15.6–20.1)	34.2	8.52*
Acanthaceae	*Bravaisia integerrima*	2 (10)	2 (10)	0.55	43.0 (41.4–44.6)	39.8 (31.8–47.7 )	0.39n.s
Caesalpiniaceae	*Dialium guianense*	3 (10)	2 (10)	0.83	24.3 (16.9–31.8)	34.5 (34.4–34.7)	−1.83n.s
FF4							
Chrysobalanaceae	*Licania platypus*	1	–	–	101	–	–
Caesalpiniaceae	*Dialium guianense*	4	–	–	60.31 (27.6–99.3)	–	–

a We indicated: (i) total number (and density, in parentheses) of trees used and available, along with the index of preference (*w_i_*); and (ii) diameter at breast height (DBH, cm) of trees used and available, along with the Student t-test for comparing differences in DBH between trees used and available. A *w_i_* value >1 indicates a positive selection; <1 indicates avoidance; and a value around 1 indicates that the sleeping trees are used proportionally to their availability. n.s. (*P*>0.05), * *P*<0.05.

b Tree availability was estimated in 0.2 ha per site (see Methods).

Most (66%) of the sleeping trees were from *Dialium guianense* (Caesalpinaceae) and *Brosimum alicastrum* (Moraceae). Based on the *w_i_* index, spider monkeys seem to select different species of sleeping trees in different sites (*B. alicastrum* in CF1 and FF1, *D. guianense* in CF3 and FF2, and *Guarea glabra* in FF3; *w_i_* >1.5 in all cases; Table 2). However, the chi-square tests were not significant (*P*>0.50, in all cases), suggesting that these species were used proportionally to its availability. Similarly, testing for differences in the DBH of trees used versus available within the home range, in most cases we did not detected significant differences in DBH (Table 2), indicating that spider monkeys did not select larger trees.

## Discussion

During the last decades, researchers have increasingly recognized that latrines of primates have important implications for seed dispersal and seedling recruitment [22]. However, the lack of information on the density and spatial distribution of sleeping sites and latrines and the characteristics of sleeping trees have hampered the understanding of their ecological significance. We demonstrate that in the Lacandona rainforest, Mexico: (i) both the density of sleeping sites and latrines did not differ between continuous forest and forest fragments; (ii) latrines were uniformly distributed across sleeping sites, but the spatial distribution of sleeping sites within the study areas was highly variable, being particularly clumped in forest fragments; (iii) latrines were located beneath only a few sleeping tree species; and (iv) sleeping trees were larger in continuous than fragmented forests.

### Density and Distribution of Sleeping Sites and Latrines

The density of sleeping sites and latrines averaged 0.25 sleeping sites and 0.30 latrines per ha, respectively but varied greatly among sites. This large variation may be related to differences in the distribution of food resources throughout the forest, and the foraging strategy of this species. Spider monkeys are one of the largest and most frugivorous Neotropical primates [25], [50], [51], and the availability of fruits is highly variable in space and time [8]. For reducing inter-patch travel costs, resource competition, and increase foraging efficiency, spider monkeys [28], [52], [53], [54] and other primate species (*Callicebus torquatus*: [55]; *Saguinus oedipus*: [56]; *Papio cynocephalus*: [57]; *Macaca nemestrina*: [58]; *Colobus vellerosus*: [59]) typically select sleeping sites located close to the available feeding areas. Moreover, because spider monkeys returns to the same sleeping trees after their foraging excursions, they have been considered typical examples of central-place foragers (*sensu* [60]) or multiple-central place foragers [8].

The higher variation of sleeping site density within the continuous forest sites (Figure 2), may be related to the larger home range in continuous forest than in fragments [61], [62], [63], and the fact that larger home ranges can be highly dynamic, varying in size among years and seasons depending on food availability [63], [64], and/or the presence of competing groups [65], [66]. Furthermore, it has been recently demonstrated that spider monkeys forage mainly in high-quality core areas (i.e., small areas of intense use within the home ranges) that tend to vary in size and spatial location along years and seasons [63]. Thus, because sleeping sites used by *Ateles* are usually located near core areas of exclusive use [63], [67], [68], it is quite possible that in the sites with lower density of sleeping sites (CF2 and CF4, Figure 2) the 30 ha we sampled within the home ranges we estimated for the years 2007 and 2008 [32], [34], [35], were temporally underused by the monkeys when we conducted the present study (2010). Thus, following the temporal and spatial variations in core areas within the home range in continuous forest, the distribution of sleeping sites within this habitat is probably more spatially and temporally dynamic in time and space than within forest fragments. Future long-term studies analyzing temporal variations in the use of sleeping trees will be valuable to accurately test this prediction.

Interestingly, the spatial distribution of latrines was particularly clumped in two forest fragments, resulting in smaller inter-latrine distances within this forest condition than within the continuous forest. Both spatial patterns can be attributed to the small home range of spider monkeys in fragments [32], and a reduced availability of large trees (and consequently overall fruit availability) in smaller fragments (e.g., Los Tuxtlas, Mexico: [31], [33]; Lacandona, Mexico: [34]). Indeed, in response to lower fruit availability in fragments, spider monkeys increase the time feeding on leaves [32], [34], which are generally available throughout the forest and along years [69]. Thus, monkeys need to travel shorter distances in fragments, and hence, they do not need to use distantly located sleeping sites. Furthermore, the forest fragments in which we found that the distribution of sleeping sites was significantly clumped (FF2 and FF3) were located next to a paved and dirt road, and hence, local people and the noise produced by cars could harass the monkeys forcing them to use the sleeping trees available in the interior of the patch.

### Characteristics of Sleeping Trees

The choice of specific sleeping trees by primates is crucial for survival, as they spend a large proportion of their time in these trees [28], [67], [68]. In this sense, we found that despite the diversity of tree species present in the Lacandona rainforest [42], spider monkeys used a small number of tree species as sleeping trees (Table 2); *Dialium guianense* and *Brosimum alicastrum* were particularly used in both continuous and fragmented forests. Both species are considered top-food species for this primate [25], [33] and are dominant and ecologically important tree species in the Lacandona rainforest [70], [71], [72]. Although spider monkeys used these species proportionally to their availability, these species and other tree species that were used (e.g., *Licania platypus, Terminalia amazonica,* and *Pouteria* sp.) shared a similar tree architecture. For example, they are all trees that reach over 40 m high, and structurally have straight trunks with large and well shaped buttresses, as well as big crowns that offer abundant and very long lateral branches [71]. This kind of tree architecture can give support and comfort in the face of adverse climatic conditions [67], [73], [74], as well as protection against predators [67], [74]. The long horizontal branches of these tree species are adequate for the locomotor suspensory pattern of spider monkeys, facilitating their movements inside and around of peripheries of crowns [75]. The fact that spider monkeys did not select larger trees for sleeping in both forest conditions could actually represent a protection strategy against adverse climatic conditions.

### Implications for Seed Dispersal and Forest Regeneration

Latrines of spider monkeys receive hundreds of seeds from a large number of plant species ([23], A. González-Zamora et al. unpublished data). In spite of potential negative effects of density-dependent mortality factors (e.g., seed/seedling predators) [76], evidence indicates that seedlings and saplings of some plants dispersed by primates recruit well at or near latrines [77], [78], [79]. Although seed/seedling aggregation in latrines can reduce the *per capita* seed-to-seedling survival [29], the large and constant arrival of seeds can produce a saturation of some biotic mortality agents (e.g., rodents, insects) [79], [80], [81], [82], permitting the recruitment and survival of seedlings and saplings within latrines [29], [79]. In fact, saplings can have higher growth rates in latrines [79]. This is probably related to the fact that latrines are enriched in nutrients compared to surrounding areas [17], [19]. Furthermore, not all seeds removed by rodents are predated, as seeds in fecal clumps may be secondarily dispersed by rodents and dung beetles, reducing the negative effects of clumping [83]. Dung can disappear quickly due to dung beetles (<3 hours: [84]; <7 hours: [83]), reducing the effect of dung on seed predation and, at the same time, potentially increasing the probability of seed burial, secondary seed dispersal by dung beetles, and seedling establishment [23], [83], [84], [85], [86].

Therefore, assuming that latrines may represent hotspots of seedling recruitment within the forest [23], [29], our results have important implications for seed dispersal and forest regeneration. First, the large variation in the spatial distribution of sleeping sites across the forest indicates that spider monkeys may create a complex seed deposition pattern in space and time. In fact, evidence indicates that this species can create a highly structured seed shadow, with a fraction of seeds deposited in individual scats distributed across the forest (scattered pattern), and a fraction deposited in sleeping sites (spatially contagious pattern) [7], [28]. This mixed seed deposition pattern can result in different areas of seedling recruitment within the forest [7], [23].

Second, because seed dispersal distance may be critical for some tree species to escape areas of high mortality (‘escape hypothesis’, see [76]), our results suggest that the effectiveness of spider monkeys as seed dispersers (*sensu* [87]) may be lower in forest fragments, in which the inter-latrine distances were notably shorter than in continuous forest, and hence, seed/seedling survival could be lower in latrines located in forest fragments. Although this hypothesis needs to be adequately tested by comparing seed/seedling/sapling survival in latrines located in continuous versus fragmented sites, Chaves et al. [32], [34] also suggests that the effectiveness of spider monkeys as seed dispersers may be limited in fragments of Lacandona forest, in which spider monkeys swallow a lower proportion of seeds and spend a higher proportion of time consuming leaves, resulting in a lower number of fecal samples containing seeds than in continuous forests.

Finally, botanists have traditionally classified tropical rainforests based on specific vegetation associations (e.g., *Terminalia*-*Dialium, Brosimum-Dialium* in southeastern Mexican rainforest [72], [88]). If as discussed above, seeds deposited in latrines by spider monkeys regenerate well [77], [78], [79], our results support the idea that seed dispersal by spider monkeys could contribute to creating these types of vegetation associations, as this primates consistently used trees of *Dialium, Brosimum,* and *Terminalia* to sleep in different sites, depositing copious amounts of seeds from different top fruit species such as *Dialium* and *Brosimum* (A. González-Zamora *et al.* unpublished data). However, because seeds must go through many subsequent filters to reach adulthood, further long-term studies evaluating seed germination and seedling and sapling establishment and survival are needed to accurately test this hypothesis.
